# Health-related quality of life after thulium fiber laser lithotripsy: a prospective study according to stone localization

**DOI:** 10.1038/s41598-026-53534-z

**Published:** 2026-06-08

**Authors:** Ali Can Albaz, Oktay Üçer, Ahmet Yılmaz, Gökhan Temeltaş, Talha Müezzinoğlu

**Affiliations:** Department of Urology, Faculty of Medicine, Manisa Celal Bayar University, 45030 Manisa, Turkey

**Keywords:** Urolithiasis, Endoscopic stone surgery, Health-related quality of life, SF-36, Stone localization, Patient-reported outcomes, Diseases, Medical research, Urology

## Abstract

To evaluate postoperative changes in health-related quality of life (HRQoL) after endoscopic stone surgery and to determine whether recovery patterns differ according to stone localization. In this prospective observational study, 50 patients undergoing endourological stone surgery were stratified according to stone localization (renal, ureteral, and bladder). HRQoL was assessed using the SF-36 questionnaire preoperatively, on postoperative day 1, and at 1, 3, and 6 months. Stone-free status was evaluated at postoperative month 1 using KUB radiography and/or non-contrast CT when clinically indicated. Statistical analysis was performed using mixed-design repeated-measures ANOVA, with appropriate assumption testing. SF-36 total scores and all subscale scores improved significantly over time in all groups (*p* < 0.001). No significant overall differences were observed between localization groups (*p* > 0.05), whereas a significant group × time interaction was detected (*p* = 0.005), suggesting differences in recovery patterns according to stone localization. Patients with ureteral stones demonstrated a faster improvement in the bodily pain domain (*p* = 0.002). Stone-free rates were high across groups (100% bladder, 94.7% ureteral, and 92.6% renal stones). Endoscopic stone surgery is associated with significant improvements in HRQoL across all stone localizations. Although recovery patterns vary according to stone location, overall functional and psychosocial outcomes improve consistently over time. However, given the observational design and lack of a comparator group, modality-specific conclusions cannot be drawn.

## Introductıon

Urinary stone disease represents a significant clinical and socioeconomic burden worldwide due to its high prevalence, recurrent nature, and long-term negative impact on quality of life and renal function^1–3^. Recent epidemiological data demonstrate a rising incidence of urolithiasis, which has been attributed to changes in dietary habits, sedentary lifestyle, increasing obesity prevalence, and metabolic disorders^1–3^. Beyond acute renal colic episodes, recurrent emergency department visits, repeated surgical interventions, and productivity loss contribute to the chronic disease burden associated with urinary stone disease^2,3^.

The anatomical location of urinary stones is a key determinant of clinical presentation, treatment strategy, and postoperative recovery. Renal stones, particularly those located in the lower pole, are associated with anatomical challenges related to calyceal configuration and infundibulopelvic angle, leading to reduced spontaneous clearance and a higher risk of residual fragments after intervention^4,5^. Ureteral stones often present with more acute symptoms; however, retropulsion and interaction with the ureteral wall during endoscopic treatment may influence surgical efficiency and postoperative outcomes^6^. In contrast, bladder stones are commonly associated with irritative lower urinary tract symptoms, and surgical removal is typically followed by a more rapid improvement in patient-reported well-being^7^. This anatomical heterogeneity suggests that stone location may influence not only technical success but also functional recovery and quality-of-life outcomes.

Endourological procedures have become the standard approach for the management of renal, ureteral, and bladder stones^8^. Advances in endoscopic equipment and laser lithotripsy technologies have significantly improved stone fragmentation efficiency and procedural safety. Although Holmium:YAG laser has long been regarded as the gold standard for laser lithotripsy, its relatively high retropulsion, requirement for larger fibers, and limited pulse modulation represent important limitations^9,10^.

The introduction of thulium fiber laser (TFL) represents a recent technological development in endourology. Owing to its higher water absorption, lower tissue penetration depth, longer pulse duration, and compatibility with ultra-thin fibers, TFL has been proposed as a next-generation laser system potentially addressing some limitations of the Holmium:YAG laser^9–11^. Recent systematic reviews, meta-analyses, and comparative clinical studies have demonstrated that TFL provides lower retropulsion and effective fragmentation and dusting performance, particularly in ureteral and lower pole renal stones, with comparable or improved operative efficiency^11–13^. However, whether these technical advantages translate into meaningful improvements in patient-reported outcomes, particularly health-related quality of life, remains to be fully clarified.

Despite these technological advances, assessing surgical success solely based on stone-free rates and complication profiles may be insufficient from a patient-centered perspective. Urinary stone disease adversely affects quality of life through pain, physical activity limitation, social dysfunction, and psychological distress^14,15^. The magnitude and timing of postoperative quality-of-life improvement may vary according to stone location and treatment modality, highlighting the importance of incorporating validated patient-reported outcome measures into clinical evaluation^14^.

In this study, health-related quality of life was assessed using the Medical Outcomes Study 36-Item Short Form Health Survey (SF-36), a well-validated and widely accepted instrument for multidimensional health status assessment^16^. Application of SF-36 in the preoperative period and during early and mid-term postoperative follow-up enables objective evaluation of functional recovery and return to daily life beyond conventional surgical outcomes.

The aim of this study was to evaluate short- and mid-term changes in health-related quality of life following endoscopic stone surgery in patients with renal, ureteral, and bladder stones, according to stone localization. This patient-centered approach aims to better define the influence of stone localization on postoperative recovery patterns and HRQoL outcomes.

## Materıals and methods

This prospective, observational study was conducted at the Department of Urology, Manisa Celal Bayar University Faculty of Medicine, and included patients evaluated for urinary stone disease who were scheduled for endourological surgical treatment. The study protocol was approved by the Manisa Celal Bayar University Health Sciences Ethics Committee (decision date: 03 April 2024; approval no: 20.478.486/2353) and was conducted in accordance with the principles of the Declaration of Helsinki and its later amendments. Written informed consent was obtained from all participants prior to enrollment.

A total of 50 consecutive adult patients were prospectively included in the study. Baseline demographic characteristics and clinical variables, including stone burden, anatomical stone localization, history of previous urinary stone surgery, prior stone treatment, and operative time, were systematically recorded before surgery. Stone burden was determined based on preoperative radiological imaging findings. Patients were categorized into three groups according to stone localization: renal, ureteral, and bladder stones. This grouping was performed to enable comparative evaluation of surgical outcomes and health-related quality of life according to anatomical stone location. Subgroup analyses involving bladder stones were considered exploratory due to the small sample size.

All patients underwent endourological treatment using a thulium fiber laser system (Quanta System, Italy), which was acquired through financial support provided by the Manisa Celal Bayar University Scientific Research Projects Coordination Unit (project no: 2024-054). The laser system operated with a maximum power output of 60 W and settings of up to 6J energy and 2500 Hz frequency. The surgical approach was determined based on stone localization and contemporary guideline-based endourological practice. Ureteral and bladder stones were treated using ureterorenoscopy, whereas renal stones were managed with retrograde intrarenal surgery. These techniques were selected because of their minimally invasive nature and favorable stone-free rates. Laser lithotripsy was performed using dusting or fragmentation techniques according to stone characteristics and surgeon preference, utilizing laser fibers ranging from 150 to 272 μm.

Postoperative assessment of residual stone status was performed in accordance with current guideline recommendations. All patients underwent plain kidney–ureter–bladder (KUB) radiography on postoperative day 1. At postoperative month 1, radiological evaluation was repeated using KUB radiography and/or non-contrast computed tomography, as clinically indicated. The choice of imaging modality was based on clinical indications, including symptom persistence and stone characteristics. Stone-free status was defined as the absence of residual stones or the presence of clinically asymptomatic residual fragments measuring ≤ 2 mm.

Health-related quality of life was assessed using the validated Short Form-36 (SF-36) questionnaire. The questionnaire was administered at five predefined time points: the day before surgery, postoperative day 1, and at postoperative months 1, 3, and 6. This repeated-measures design enabled objective evaluation of both short- and mid-term changes in quality of life following surgical treatment. All available data were included in the analysis; no imputation method was applied for missing data.

Patients younger than 18 years of age, those with active urinary tract infection, stones measuring ≥ 15 mm, ureteral strictures, acute renal failure, pyonephrosis, a history of open ureteral surgery, or congenital urinary tract anomalies (such as horseshoe kidney) were excluded from the study to ensure a homogeneous study population and to improve the reliability of outcome assessment.

Statistical analyses were performed using IBM SPSS Statistics, version 26.0 (IBM Corp., Armonk, NY, USA). The distribution of continuous variables was assessed using the Kolmogorov–Smirnov test.

To evaluate longitudinal changes in health-related quality of life and differences between stone localization groups, a mixed-design repeated-measures ANOVA was applied^17^, with time as the within-subject factor and stone localization as the between-subject factor. Assumptions of normality and sphericity were assessed, and when the sphericity assumption was violated, Greenhouse–Geisser correction was applied.

For baseline comparisons between groups, non-parametric tests were used due to small subgroup sizes. The Kruskal–Wallis test was used for overall comparisons, followed by Mann–Whitney U tests with Bonferroni correction for pairwise comparisons when appropriate.

Categorical variables were analyzed using the chi-square test or Fisher’s exact test, as appropriate. All statistical tests were two-tailed, and *p* < 0.05 was considered statistically significant.

## Results

### Patient characteristics and perioperative outcomes

A total of 50 consecutive patients were prospectively included in the study and stratified according to stone localization as bladder (n = 4), ureteral (n = 19), and renal stones (n = 27). Baseline demographic and perioperative characteristics of the study population are summarized in Table 1. No statistically significant differences were observed between groups for age, sex distribution, stone volume, operative time, or laser activation time (*p* > 0.05 for all).Stone-free rates assessed at postoperative month 1 were high across all groups, reaching 100% in bladder stones, 94.7% in ureteral stones, and 92.6% in renal stones, with no statistically significant difference between groups (*p* > 0.05).

**Table 1:** Baseline demographic and perioperative characteristics according to stone localization.

Variable	Bladder stones (n = 4)	Ureteral stones (n = 19)	Renal stones (n = 27)	*p* value
Age, years	58.5	47.0	42.0	> 0.05
Sex (male/female)	4 / 0	11 / 8	15 / 12	> 0.05
Stone volume, mm^3^	2750	1100	2300	> 0.05
Operative time, min	55	45	70	> 0.05
Laser activation time, min	16	9	11	> 0.05
Stone-free rate at postoperative month 1 (%)	100	94.7	92.6	> 0.05

Stone-free status was defined as the absence of residual stones or the presence of asymptomatic fragments ≤ 2 mm.

### Health-related quality of life outcomes

#### Overall SF-36 score

**Fig. 1:**
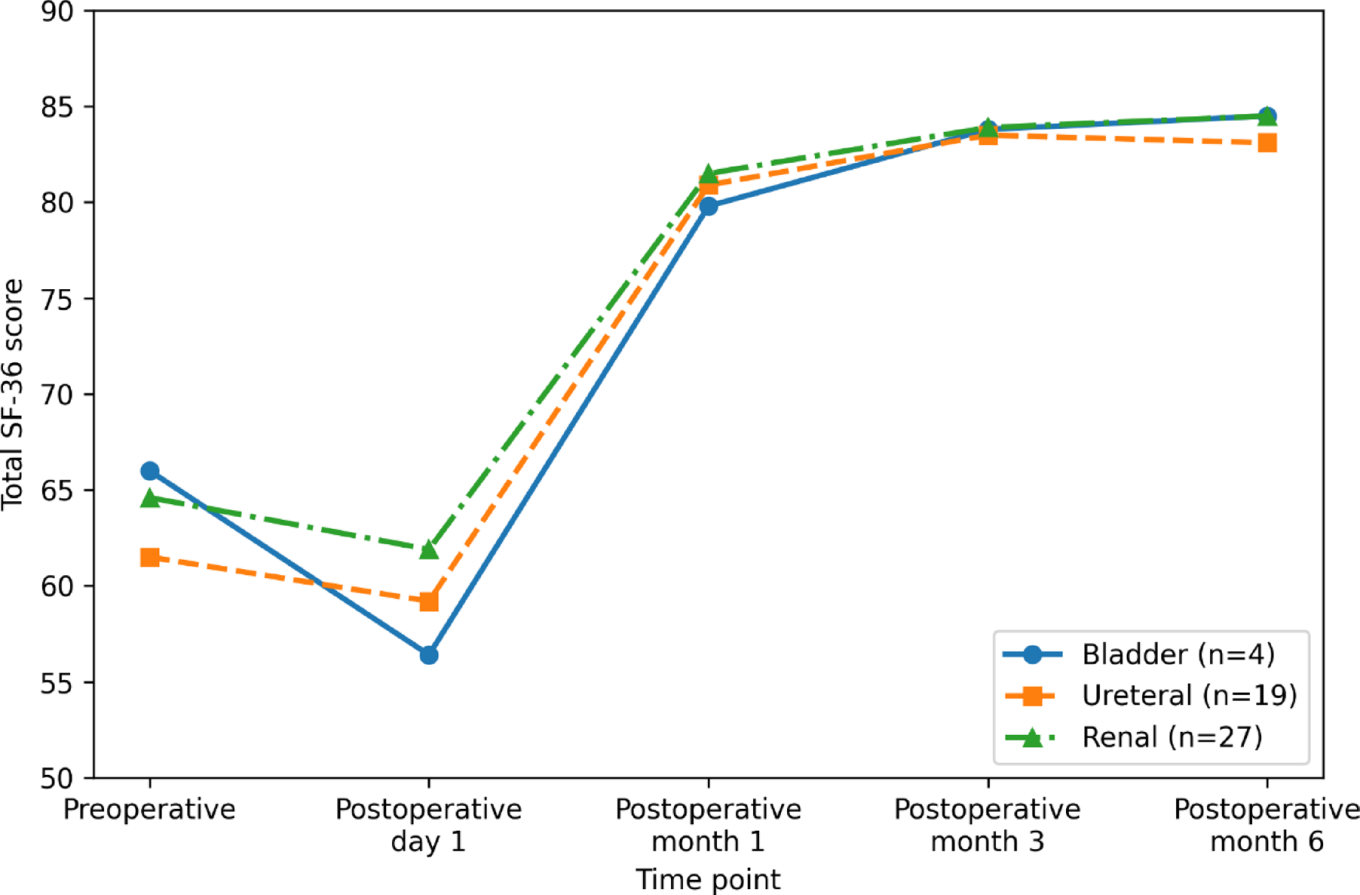
Changes in total SF-36 scores over time according to stone localization. Total SF-36 scores showed a significant improvement over time in all stone localization groups following endourological treatment. Mixed-design repeated-measures ANOVA demonstrated a significant main effect of time (*p* < 0.001), indicating progressive postoperative improvement in overall health-related quality of life. The main effect of group was not statistically significant (*p* = 0.110); however, a significant group × time interaction was observed (*p* = 0.005), suggesting differences in the temporal pattern of recovery according to stone localization. Data are presented as mean values.

Total SF-36 scores improved significantly over time in all three stone localization groups (Fig. 1).

Mixed-design repeated-measures ANOVA demonstrated a significant main effect of time (*p* < 0.001), indicating a consistent postoperative improvement in overall quality of life. The main effect of group was not statistically significant (*p* = 0.110), whereas a significant group × time interaction was observed (*p* = 0.005), suggesting differences in the temporal pattern of quality-of-life improvement according to stone localization.

### Physical health domain

**Fig. 2:**
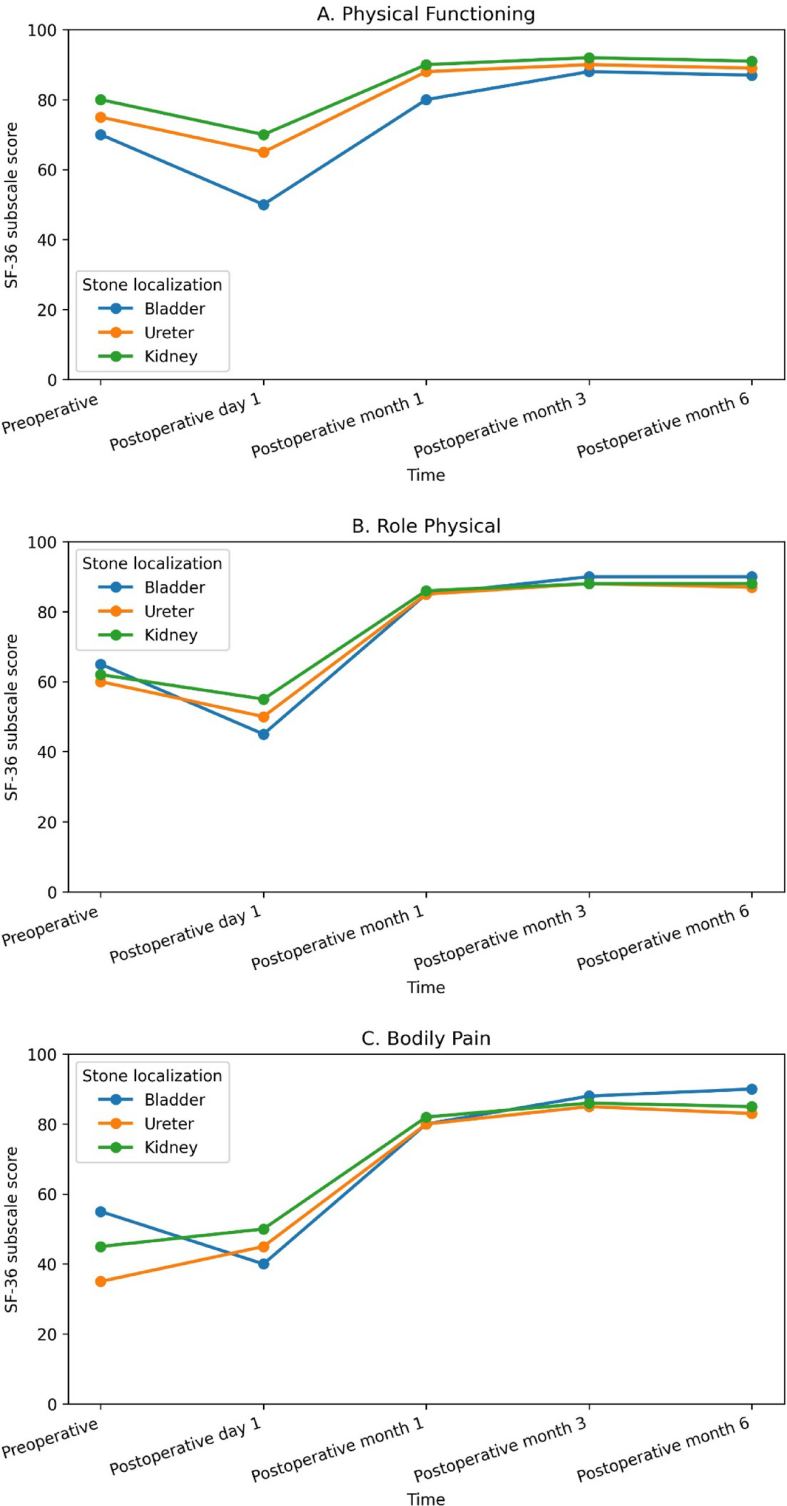
Physical health–related SF-36 subdomains according to stone localization. Changes over time in physical health–related SF-36 subdomains are shown for each stone localization group: (**A**) Physical Functioning, (**B**) Role Limitations due to Physical Health, and (**C**) Bodily Pain. All physical subdomains demonstrated significant postoperative improvement over time (main effect of time, *p* < 0.001 for all). While Physical Functioning and Role Physical scores improved similarly across groups, Bodily Pain exhibited a significant group × time interaction (*p* = 0.002), indicating localization-dependent differences in pain-related recovery. Data are presented as mean values.

Physical health–related SF-36 subdomains are presented in Fig. 2.

Physical functioning and role limitations due to physical health improved significantly over time in all groups, with a significant main effect of time (*p* < 0.001) and no significant group or group × time interaction.

Bodily pain scores also improved significantly over time (*p* < 0.001), and a significant group × time interaction was observed (*p* = 0.002), indicating differences in pain-related recovery according to stone localization.

### Psychosocial health domain

**Fig. 3:**
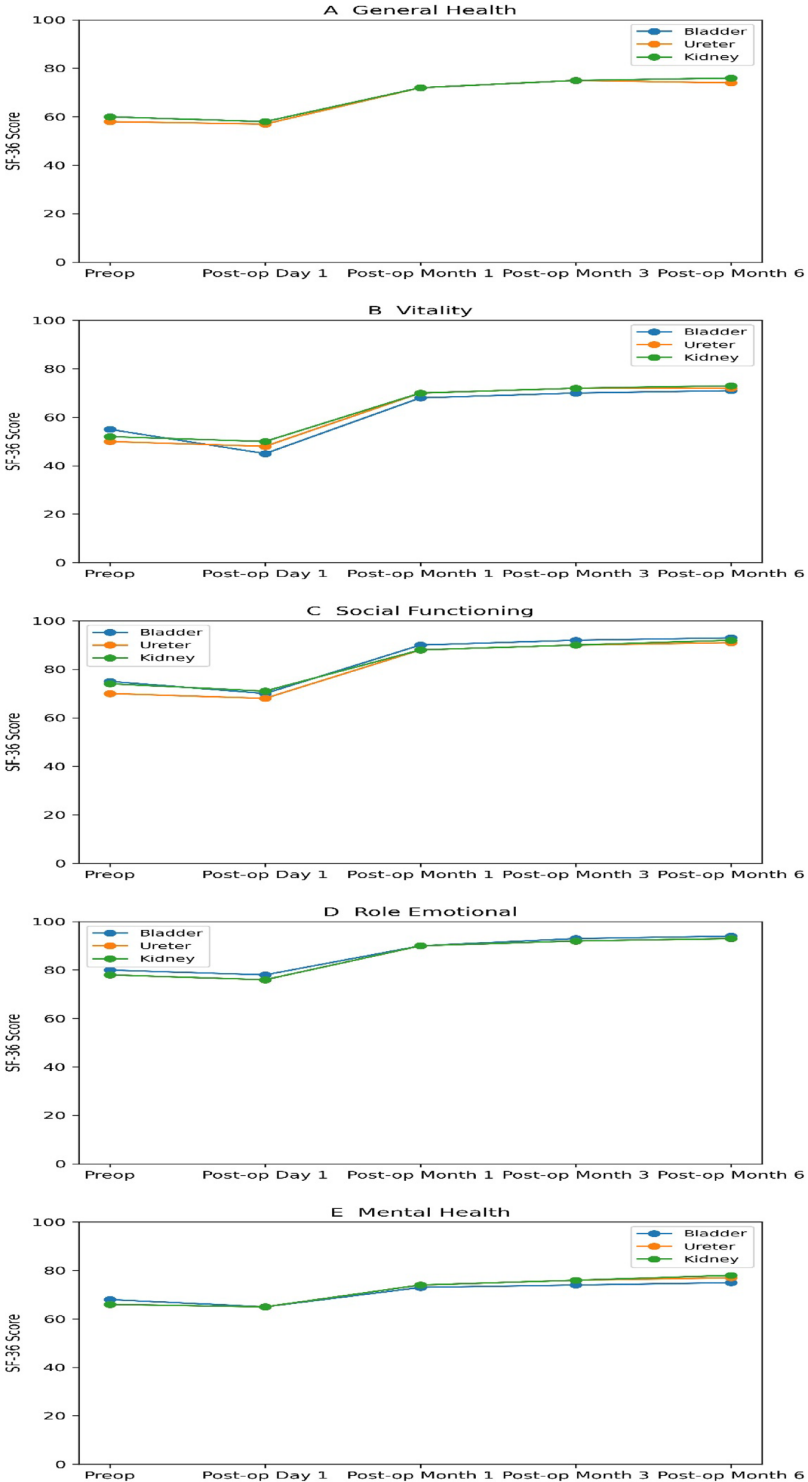
Psychosocial health–related SF-36 subdomains according to stone localization. Temporal changes in psychosocial SF-36 subdomains are illustrated for each stone localization group: (**A**) General Health, (**B**) Vitality, (**C**) Social Functioning, (**D**) Role Limitations due to Emotional Problems, and (**E**) Mental Health. All psychosocial domains showed significant improvement over time (main effect of time, *p* < 0.001 for all). A significant main effect of group was observed for Role Emotional (*p* = 0.005), whereas no significant group × time interactions were detected for psychosocial subdomains, indicating comparable recovery trajectories across stone localizations. Data are presented as mean values.

Psychosocial SF-36 subdomains are illustrated in Fig. 3. General health perception and vitality scores increased significantly over time in all groups, with a significant main effect of time (*p* < 0.001) but no significant group or interaction effects.

Social functioning and emotional role limitation subdomains similarly demonstrated significant postoperative improvement. Notably, emotional role limitation exhibited a significant main effect of group (*p* = 0.005), suggesting overall differences between stone localization groups, although the group × time interaction was not significant.

Mental health scores improved significantly over time in all three groups, with no statistically significant differences between groups or interaction effects.

## Dıscussıon

For many years, surgical success in urinary stone disease has primarily been assessed using technical parameters such as stone-free rates, complication profiles, and operative time^18,19^. However, with the rapid evolution of endourological techniques and laser technologies, there is growing emphasis that surgical effectiveness should not be confined to procedural success alone, but should also be evaluated in conjunction with patient-centred outcomes^20,21^. In particular, rapid functional recovery and timely return to daily activities—key objectives of minimally invasive stone surgery—have rendered health-related quality-of-life (HRQoL) measurements a complementary component of treatment success^20,21^. Nevertheless, the number of studies prospectively and comparatively examining the impact of laser technologies on HRQoL specifically according to stone anatomical location remains limited.

In the present study, we prospectively evaluated the effects of endourological stone surgery performed with thulium fiber laser (TFL) on HRQoL in patients with bladder, ureteral, and renal stones using a multi-time-point design. Our findings demonstrate that endourological stone surgery yields significant improvements in the SF-36 total score and across all subdomains, irrespective of stone location. These results suggest that endourological stone surgery may support a patient-centred treatment approach by promoting postoperative functional well-being.

The technical advantages of TFL have been comprehensively described in the literature. Low retropulsion, allowance for smaller-calibre fibres, stable energy delivery at high frequencies, and limited thermal injury position TFL favourably in terms of both efficacy and safety^9,22–24^. The high stone-free rates observed in our cohort (92.6–100%) further support that these technical strengths translate into clinical outcomes. Importantly, our findings suggest that the benefits extend beyond operative success and are also reflected in patient-reported outcomes such as HRQoL.

One of the most notable findings was the significant group × time interaction for both the SF-36 total score and the pain subdomain. This suggests that, although HRQoL improved across all stone locations, the temporal pattern of recovery may vary according to anatomical site. In particular, the earlier and more pronounced pain-related HRQoL improvement in patients with ureteral stones may be explained by the more acute and severe symptom profile typically associated with ureteral calculi^25–28^. Surgical relief of ureteral obstruction can rapidly alleviate pain perception, which may be strongly reflected in HRQoL scores during the early postoperative period^21,25,28^.

In contrast, the more indolent or chronic course of symptoms in bladder and renal stones may have resulted in a more gradual and balanced trajectory of HRQoL improvement^29,30^. Nevertheless, the convergence of HRQoL to similar levels across groups at longer follow-up indicates that endourological stone surgery is effective in terms of ultimate patient outcomes regardless of stone location^21^. Collectively, these findings imply that stone location may influence the pace of recovery but does not appear to limit long-term functional gains.

The absence of a significant group × time interaction in other SF-36 subdomains—such as physical functioning, role limitations due to physical problems, vitality, social functioning, and mental health—also carries clinical relevance. This pattern suggests that the minimally invasive nature of TFL and its low degree of tissue trauma may enable comparable functional and psychosocial recovery across anatomical locations^31–33^. Although operative outcomes of laser technologies have been frequently reported, data addressing social and psychological well-being remain relatively limited^14^. This gap hampers a clear understanding, during clinical decision-making, of how technical success relates to the patient’s perceived well-being. Yet, the true success of surgical treatment should be judged not only by objective procedural outcomes but also by how these outcomes translate into daily life and psychosocial functioning. In this context, our study provides a patient-centred contribution to the literature by demonstrating meaningful postoperative improvements not only in the physical components of HRQoL but also in psychosocial domains.

The finding of a significant main effect of group—without a significant group × time interaction—in the role-emotional subdomain suggests that this parameter may be more sensitive to baseline group characteristics than to the speed of postoperative improvement. Role-emotional scores may be influenced less by stone location and more by the patient’s psychological status, treatment expectations, and social support mechanisms. Therefore, when interpreting between-group differences in role-emotional outcomes, psychosocial variables should be considered alongside biomedical factors.

The lack of significant differences in demographic and perioperative variables across groups further strengthens the internal validity of our findings. The homogeneity of the groups with respect to age, sex, stone burden, and operative time suggests that changes in HRQoL were largely driven by surgical treatment and the time factor. Moreover, the similarly high stone-free rates across all groups support that endourological stone surgery is an effective treatment approach for both upper and lower urinary tract stones^9^.

A major strength of this study is that HRQoL was assessed prospectively at multiple time points rather than at a single time point. This approach captures not only the magnitude of postoperative improvement but also its temporal dynamics^21^. In addition, the use of a mixed-design repeated-measures ANOVA enabled simultaneous evaluation of time and group effects, thereby enhancing the statistical power of the analysis^17^.

Nevertheless, several limitations should be acknowledged. First, this prospective study reflects a real-world consecutive cohort of patients undergoing endourological stone surgery within a predefined study period. Therefore, the sample size was determined by the number of patients presenting with appropriate surgical indications during this timeframe rather than by predetermined recruitment targets. In particular, the smaller number of bladder stone cases likely reflects the lower clinical incidence of bladder calculi compared with ureteral and renal stones reported in the literature^30^. Second, the study did not include a comparison arm using alternative laser technologies such as Holmium:YAG, which precludes direct comparative evaluation of different lithotripsy modalities. Third, although disease-specific quality-of-life instruments for urolithiasis have been proposed, the SF-36 questionnaire was selected because it is a widely validated and internationally accepted instrument for assessing multidimensional health-related quality of life in surgical populations. Finally, the follow-up period was limited to six months, and therefore longer-term HRQoL trajectories warrant further investigation. Potential confounding factors such as stone composition, ureteral stenting, analgesic use, and patient comorbidities were not systematically controlled and may have influenced HRQoL outcomes.

In conclusion, our results indicate that endourological stone surgery was associated with significant and sustained improvement across all dimensions of HRQoL, irrespective of stone location. While patients with ureteral stones demonstrated a more rapid improvement in pain and overall HRQoL, similar recovery patterns were observed across groups in physical and psychosocial domains over time. These findings highlight the clinical effectiveness of endourological stone surgery and underscore the importance of integrating patient-reported outcomes into the evaluation of treatment success in stone surgery.

## Conclusıon

This prospective study demonstrates that endourological stone surgery was associated with significant and sustained improvements in health-related quality of life, irrespective of stone localization. Across bladder, ureteral, and renal stone groups, postoperative improvements were observed in total SF-36 scores as well as in both physical and psychosocial domains.

Although quality-of-life improvement was evident in all groups, recovery dynamics varied according to stone localization, with earlier pain relief observed in patients with ureteral stones. In contrast, similar recovery trajectories across other quality-of-life domains suggest a generalized return to daily activities independent of anatomical location.

The consistently high stone-free rates across all groups further support the effectiveness of endourological stone surgery. These findings highlight the importance of incorporating patient-reported outcomes alongside radiological success when evaluating treatment effectiveness in stone surgery.

## Data Availability

The datasets generated and/or analyzed during the current study are available from the corresponding author on reasonable request.
